# Arecoline-Induced Hepatotoxicity in Rats: Screening of Abnormal Metabolic Markers and Potential Mechanisms

**DOI:** 10.3390/toxics11120984

**Published:** 2023-12-04

**Authors:** Jing Sun, Kai Zhang, Yihui Yin, Yunpeng Qi, Siyuan Li, Haonan Sun, Min Luo, Yixuan Sun, Zhiying Yu, Jie Yang, Jingjing Wu, Lijuan Chen, Wenjuan Xu, Ling Dong

**Affiliations:** 1 School of Life Sciences, Beijing University of Chinese Medicine, Beijing 102488, China; 20210935063@bucm.edu.cn (J.S.); zk006086@163.com (K.Z.); 20220941106@bucm.edu.cn (Y.Y.); 13054821866@163.com (Y.Q.); bucmlsy@126.com (S.L.); 13728881200@163.com (H.S.); sunyixuan2000@126.com (Y.S.); yuzhiying2020@163.com (Z.Y.); clj18380983046@163.com (L.C.); 2 School of Chinese Materia Medica, Beijing University of Chinese Medicine, Beijing 102488, China; luomin26@outlook.com (M.L.); forsythia0227@163.com (J.Y.); a3175522168@163.com (J.W.)

**Keywords:** Arecoline, hepatotoxicity, metabolomics, network toxicology

## Abstract

Arecoline is a pyridine alkaloid derived from areca nut in the Arecaceae family. It has extensive medicinal activity, such as analgesic, anti-inflammatory, and anti-allergic. However, the toxicity of Arecoline limits its application. Most current studies on its toxicity mainly focus on immunotoxicity, carcinogenesis, and cancer promotion. However, there are few systematic studies on its hepatotoxicity and mechanisms. Therefore, this research explored the mechanism of hepatotoxicity induced by Arecoline in rats and analyzed endogenous metabolite changes in rat plasma by combining network toxicology with metabolomics. The differential metabolites after Arecoline exposure, such as D-Lysine, N4-Acetylaminobutanal, and L-Arginine, were obtained by metabolomics study, and these differential metabolites were involved in the regulation of lipid metabolism, amino acid metabolism, and vitamin metabolism. Based on the strategy of network toxicology, Arecoline can affect the HIF-1 signaling pathway, MAPK signaling pathway, PI3K-Akt signaling pathway, and other concerning pathways by regulating critical targets, such as ALB, CASP3, EGFR, and MMP9. Integration of metabolomics and network toxicology results were further analyzed, and it was concluded that Arecoline may induce hepatotoxicity by mediating oxidative stress, inflammatory response, energy and lipid metabolism, and cell apoptosis.

## 1. Introduction

Arecoline is a class of pyridine alkaloids extracted from areca nut, which has good biological activity and potential application value. Arecoline is one of the main active ingredients of areca nut, with anti-parasitic, promoting gastrointestinal digestion, analgesia, anti-inflammatory, anti-allergy, and other effects [1,2,3]. It is widely used in clinical practice in taeniasis, abdominal pain, and malaria. Areca nut has been used for thousands of years. At present, about 700 million people in the world have the habit of chewing areca nuts. However, excessive use can also cause different degrees of toxic reactions, such as immunotoxicity, carcinogenic and carcinogenic, reproductive toxicity, and genotoxicity [4,5,6]. Therefore, The International Agency for Research on Cancer (IARC) has classified areca nut as a Group I carcinogen. Considering the wide application of areca nut in food and clinics, it is necessary to analyze further and verify the toxicity mechanism to ensure the safety and reliability of its application.

In recent years, scholars around the world have been aware of the safety issues of areca nut and have studied its toxicity in the mouth, heart, kidney, and other aspects [7,8,9], but, until now, the molecular mechanism of its hepatotoxicity is still not fully explored. The liver is the main target organ for drug metabolism, and hepatotoxicity is a common adverse reaction of drugs and a key investigation index for drug safety assessment. Arecoline has clinically shown adverse reactions in the liver, such as cirrhosis and acute liver injury. Studies have shown that pathological changes in the ultrastructure of liver cells were found after the mice intragastric administration of areca extract [10], suggesting that the areca line had hepatotoxicity. It has also been reported that areca nuts can increase the risk of liver cancer in hepatitis virus carriers [11]. In addition, most of the existing studies on the mechanism of Arecoline hepatotoxicity are mostly concentrated at the molecular level of cell growth inhibition, induced DNA damage, and oxidative stress. Studies have found that Arecoline can cause DNA damage and induce hepatocyte apoptosis, promote the blockage of the G0/G1 cell cycle, and inhibit normal hepatocyte proliferation to induce cancer suppressor gene P53 phosphorylation, leading to the carcinogenesis of hepatocytes [12]. However, there are few reports on the relationship between Arecoline metabolism and hepatotoxicity, and its potential mechanism needs to be further studied.

Metabolomics, as an important part of systems biology, is an analytical method to study the qualitative, quantitative, or dynamic changes of endogenous small molecules produced by biological systems after external stimuli or disturbances and to describe the whole of the endogenous metabolites of living organisms and their patterns of response to changes in endogenous and exogenous factors [13,14]. Metabolomics provides a new method for elucidating the toxic mechanism of traditional Chinese medicine (TCM) by dynamically detecting the changes in metabolic profile in vivo and determining toxic targets, processes, and biomarkers [15,16,17]. Recently, LC-MS has quickly become the mainstream platform for metabolic group analysis [18,19,20]. UPLC-QE-Orbitrap-MS-based metabolomics has great potential for providing more accurate information for toxicity assessment and molecular mechanisms because of the features of scanning speed, high resolution, mass accuracy, and high sensitivity [21]. Network toxicology is an approach derived from network pharmacology, through the construction of a “drug–toxic–gene–target” network model, to clarify the potential mechanism of compound action from multiple perspectives and to identify pathways associated with the toxic target of a compound at the toxicological level [22,23]. The obtained endogenous metabolites and potential toxicity targets can elucidate the toxicity mechanism from different levels, which is a new strategy to comprehensively study the toxic mechanism of TCM.

Based on the above research status, this study will deeply explore the mechanism of Arecoline-induced hepatotoxicity on the co-application of metabolomics and network toxicology, and assess the changes in serum metabolite levels in model animals before and after Arecoline administration based on the systematic metabolomics approach of UPLC-QE-Orbitrap-MS to search for the potential hepatotoxicity-related metabolites and metabolic pathways. An amount of 28 biomarkers were found in the serum of rats exposed to arecoline. Then, a comprehensive network toxicology study was conducted based on the virtual simulation strategy. It was found that the hepatotoxicity of Arecoline may be closely related to oxidative stress, inflammatory response, energy and lipid metabolism, and cell apoptosis. This study provides data support for the study of the hepatotoxicity of Arecoline and provides a reference for the scientific development and reasonable application of areca nut.

## 2. Materials and Methods

### 2.1. Materials and Animals 

#### 2.1.1. Instrument 

UPLC-QE Orbitrap-MS (Thermo Fisher Scientific, Waltham, MA, USA); automatic biochemical analyzer (Beckman Coulter, Inc., Pasadena, CA, USA); Dalong MX-S Vortex Mixer(DLAB Scientific Co., Ltd., Bejing, China); Centrifuge 5424R refrigerated centrifuge (Eppendorf AG, Hamburg, Germany); SQP-type 1/10,000 electronic balance (Sartorius, Gottingen, Germany); and SB25-12 DTD Ultrasonic Cleaning Machine (Ningbo Xinzhi Biotechnology Co., Ltd., Ningbo, China).

#### 2.1.2. Reference Standards and Reagents

Arecoline hydrobromide (Lot: ST11839, Shanghai Shidande Standard Technical Service Co., Ltd.,Shanghai, China); 4% paraformaldehyde solution (Biosharp, Shanghai, China); sodium chloride injection (Shijiazhuang Siyao Co., Ltd., Shijiazhuang, China); mass spectrometric formic acid (Thermo Fisher Scientific, Waltham, MA, USA); and mass spectrometric acetonitrile (Thermo Fisher Scientific, USA).

#### 2.1.3. Animal Treatment

The experimental animals were purchased from Sibei Fu Experimental Animals Technology Co., Ltd. (Beijing, China) with license number “SCXK (Jing) 2019-0010”. Thirty male SD rats weighing 180–200 g were raised in an SPF-level animal laboratory of the Beijing University of Traditional Chinese Medicine. The rats were housed under the following conditions: 7 days, with an ambient temperature of 23 ± 2 °C and humidity of 35 ± 5%, day and night alternation for 12 h, unlimited drinking, and food freely fed. The rats were randomly divided into three groups, and the groups, doses, administration modes, and times are shown in Table 1.

All experiments were carried out in accordance with Chinese national legislation and local guidelines. The animal experiments were approved by the Animal Ethics Committee of the Beijing University of Traditional Chinese Medicine (BUCM-4-2021061702-2068).

### 2.2. Manifestation of Hepatotoxicity Induced by Arecoline 

#### 2.2.1. Animal Status (Behavioral) Analysis 

The body weight of the rats was closely monitored during the experiment, and the dosage was calculated based on body weight, and observe the condition of the rats before and after administration.

#### 2.2.2. Serum Biochemical Indexes Detection 

Rats were exposed continuously for 7 days. Before the last administration, all rats were fasted for 12 h with access to water. Abdominal aortic blood samples were collected while all animals were anesthetized with 20% urethane at a dose of 5 mL·kg^−1^. The blood samples were centrifuged at 3000 r·min^−1^ at 4 °C for 15 min, and the supernatant was centrifuged at 3500 r·min^−1^ at 4 °C for 10 min. Then, we extracted the supernatant. The serum was collected for subsequent serum biochemical detection and metabolomics study. The contents of Aspartate Transaminase (AST) and Alanine aminotransferase (ALT) in serum were measured using a fully automated biochemical instrument.

#### 2.2.3. Histopathological Evaluation 

We removed the rat liver and washed it with normal saline to remove the blood stains, and the surface water was removed with filter paper. The liver was immediately fixed in a 4% paraformaldehyde solution and made into paraffin sections. The pathological features of the liver were examined by hematoxylin and eosin (HE) staining. Histopathological changes were observed under the microscope.

### 2.3. Metabolomics Study on Hepatotoxicity Induced by Arecoline 

#### 2.3.1. Sample Preparation 

We selected the high-dose group of Arecoline with more severe liver injury and conducted a metabolomics study.

##### Serum Sample Preparation

After thawing the serum sample, 150 μL of serum was taken, and 450 μL of acetonitrile was added to precipitate protein, and ultrasonicated in a cold water bath for 10 min, with vortex mixed for 1 min, centrifuged at 13,000 r·min^−1^ at 4 °C for 15 min. The supernatant was dried with nitrogen, then redissolved with 75 μL 70% acetonitrile, centrifuged at 13,000 r·min^−1^ for 15 min at 4 °C, and the supernatants were used for the metabolomic analysis.

##### QC Sample Preparation

The serum of all rats was taken in equal amounts and mixed evenly. QC samples were prepared according to the preparation method of serum samples.

#### 2.3.2. Analysis Conditions 

##### Chromatographic Conditions

Serum samples (3 μL) were injected into a C18 chromatographic column (2.1 mm × 100 mm, 1.8 µm). The column temperature was set to 30 °C, the sample plate temperature was set to 4 °C, and the flow rate was set to 0.3 mL·min^−1^. The UPLC separation system includes mobile phase A (0.1% formic acid water) and mobile phase B (acetonitrile). The gradient profiles were as follows: 0–5 min, 4–30% B; 5–7 min, 30–37% B; 7–11 min, 37–60% B; 11–22 min, 60–100% B; 22–22.5 min, 100% B; 22.5–23.5 min, 4% B; 23.5–27 min, 4% B.

##### Mass Spectrometry Conditions

Using HESI ion source, positive and negative ion detection mode. The MS parameters were as follows: Fourier high-resolution scanning range of *m*/*z*, 100~1500; primary scanning resolution, 35,000; ion source temperature, 350 °C; ionization source voltage, 4 KV; capillary voltage, 35 V; tube lens voltage, 110 V; sheath gas and auxiliary gas are high purity nitrogen (purity > 99.99%), sheath gas flow rate is 12 L·min^−1^, auxiliary gas flow rate is 6 L·min^−1^ and the normalized collision energy is 20 eV, 30 eV, 40 eV.

#### 2.3.3. Methodological Investigation 

##### Instrument Precision Test

The same QC sample was taken and injected into the mass spectrometer 6 times. According to the results obtained, the peak area was extracted, and the RSD value of the peak area of each ion was calculated. These peak areas with RSD less than 30% should account for more than 80% of the total [24].

##### Method Reproducibility Test

According to the method of preparing serum samples, 6 identical samples were prepared and injected into the mass spectrometer at the same time to extract the peak area of each sample. These peak areas with RSD less than 30% should account for more than 80% of the total.

##### Sample Stability Test

The same QC sample solution was inserted into the whole sampling sequence at 6 time points and injected into the mass spectrometer for sampling analysis. The peak area was extracted from the obtained data. Ions with RSD < 30% should account for more than 80%. These peak areas with RSD less than 30% should account for more than 80% of the total.

#### 2.3.4. Data Processing

The peak automatic integration and extraction of peak intensity and nonlinear correction in the time domain of LC-MS data were processed by MS-DIAL 4.70 software(University of California, Davis, CA, USA). Subsequently, the data was analyzed by Xcalibur 4.2 (Thermo Fisher Scientific, Waltham, Massachusetts, USA) workstation to control ppm < 5. The compound was identified using the HMDB online database based on the accurate ion mass and characteristic ion fragments obtained from mass spectrometry. The data were imported into SIMCA14.1 software(Sartorius, Gottingen, Germany) for multivariate statistical analysis. The model was established using principal component analysis (PCA) and orthogonal partial least squares-discriminant analysis (OPLS-DA). The experimental data were analyzed using IBM SPSS Statistics 20(IBM, Armonk, New York), and an independent sample T-test or non-parametric test was performed between groups, with a statistical difference at *p* ≤ 0.05. Finally, metabolites with VIP ≥ 1 and *p* ≤ 0.05 were used as differential metabolites. Pathway enrichment was performed by MetaboAnalyst 5.0 (www.metaboanalyst.ca, accessed on 25 September 2023).

### 2.4. Network Toxicology Study on Arecoline-Induced Hepatotoxicity 

#### 2.4.1. Acquisition of Arecoline Targets 

Firstly, potential targets of Arecoline were directly retrieved from TCMSP (https://old.tcmsp-e.com/tcmsp.php, accessed on 10 September 2023) and Herb (http://herb.ac.cn/, accessed on 10 September 2023). The 2D structure of Arecoline was downloaded from PubChem (https://pubchem.ncbi.nlm.nih.gov/, accessed on 10 September 2023) and stored in SDF format. Upload the structure of Arecoline to PharmMapper (http://www.lilab-ecust.cn/pharmmapper/submitfile.html, accessed on 10 September 2023) and SwissTargetPrediction (http://www.swisstargetprediction,ch/, accessed on 10 September 2023) for potential targets. Subsequently, the obtained non-standard gene names were input into the UniProt database (http://www.uniprot.org/, accessed on 10 September 2023) and transformed into standard gene names. The genes obtained from the four databases were integrated and deduplicated to obtain Arecoline targets.

#### 2.4.2. Acquisition of Hepatotoxicity Targets 

Through PubMed (https://www.ncbi.nlm.nih.gov/, accessed on 10 September 2023), this study retrieved the keywords “liver injury” in order to obtain comprehensive hepatotoxicity-related keywords as much as possible. Then, CTD (http://ctdbase.org/, accessed on 10 September 2023) and Genecards (http://www.genecards.org/, accessed on 10 September 2023) were consulted to search for keywords one by one. After obtaining the genes related to hepatotoxicity in the CTD and genecards databases, genes with a “marker/mechanism” marker (CTD) and with a score > 40 (genecards) were selected, respectively. On the other hand, the gene expression profile chip data related to hepatotoxicity was downloaded from the GEO database, imported into the R language for analysis, cleaned and formatted the data, and translated the gene probe into the standard gene name. Finally, the differentially expressed genes were obtained by the limma package of the linear regression model, and the settings were *p* < 0.05, log FC > 2. All genes were integrated and deduplicated and the results were hepatotoxicity-related targets.

#### 2.4.3. Construction of Common Targets 

The above target information was imported into Draw Venn Diagram (https://bioinformatics.psb.ugent.be/webtools/Venn/, accessed on 10 September 2023), and the common target was obtained by resulting from the intersection of Arecoline targets and hepatotoxicity targets, that is, the direct target of Arecoline-induced hepatotoxicity in rats.

#### 2.4.4. Protein–Protein Interaction (PPI) Network Construction 

Common targets of Arecoline were imported into the string database (https://String db.org/, accessed on 10 September 2023) and the results of PPI were downloaded and imported into Cytoscape 3.8.2 software for visualization. Topological analysis of the PPI Network is performed using the “Network Analyzer” function. In network analysis, the size and color of nodes depend on the size of the degree value. The larger the degree value, the larger the node, and the darker the color. The strength of two nodes determines the width of the edge, and the stronger their interaction, the wider the edge.

#### 2.4.5. GO Bioprocess Analysis and KEGG Enrichment Analysis

To further investigate the functions and enrichment pathways of these toxic genes, GO bioprocess analysis and KEGG enrichment analysis were performed by the DAVID database (https://david.ncifcrf.gov/, accessed on 10 September 2023). And “enrichment.component”, “enrichment.Function”, “enrichment.Process” and “enrichment.KEGG” as obtained data can be visualized with Omicshare (http://www.omicshare.com/tools/index.php/, accessed on 10 September 2023).

## 3. Results

### 3.1. Manifestations of Hepatotoxicity Induced by Arecoline

#### 3.1.1. Animal Status (Behavioral) Analysis 

**Figure 1:**
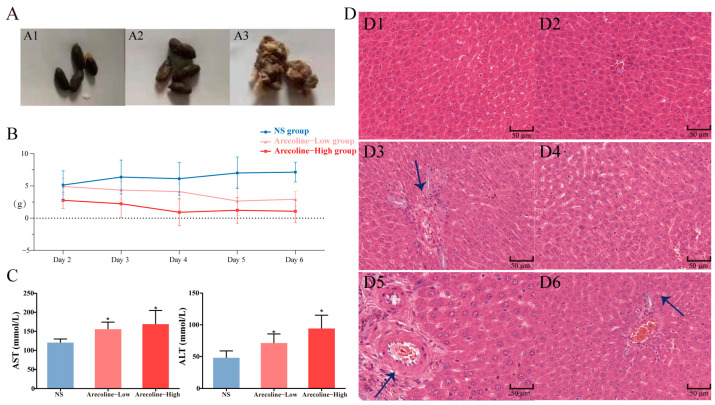
(**A**) Fecal characters of rats in each group. n = 6, NS group (**A1**), Arecoline—low group (**A2**), Arecoline—high group (**A3**). (**B**) The body weight gain of rats in the NS group and the Arecoline group within 6 days. n = 6. (**C**) Serum AST and ALT levels in NS group and Arecoline group (n = 6, compared with NS group: * p < 0.05). (**D**) Histopathological changes of liver induced by Arecoline in rats(Arrows indicate inflammatory cells or fat vacuoles). NS group (**D1**,**D2**), Arecoline—low dose group (**D3**,**D4**), Arecoline—high dose group (**D5**,**D6**).

After giving the drugs to each group, no significant abnormalities were observed in the NS group. Compared with the NS group, the Arecoline group showed varying degrees of reduced physical activity, curled up in the corners of the cage, decreased food intake, sluggish response to external stimuli (sounds), and dull fur symptoms. Some Arecoline-exposed rats presented with generalized seizures and redness of the nose. At the end of the dosing period, the anus of Arecoline group rats was red and swollen, and the buttocks and tail showed different degrees of loose stool, which was deposited on the surface of animal fur. We collected feces from different groups (Figure 1A) and observed some animal’s self-directed behavior. There was a significant difference between the NS group and some high-dose rats. Poisoned animals would turn or half turn aimlessly, which may be due to infection after weight loss. As of the last day, three rats in the high-dose group died, while there were no deaths in the other groups. During the dosing period, the weight gain of rats in the Arecoline group slowed down (Figure 1B), but the liver became congested after dissection.

#### 3.1.2. Analysis of Serum Biochemical Indexes 

AST and ALT are classic liver injury detection indexes, and their contents reflect the damage of drugs to target organs to a certain extent. This study quantitatively detects their contents to assess the degree of liver injury induced by Arecoline in rats. The results showed that after Arecoline administration, as shown in Figure 1C, the levels of ALT and AST in the rat serum were higher than those of the NS group (*p* < 0.05).

#### 3.1.3. Histopathological Evaluation

Histopathological sections are generally considered routine for toxicity evaluation. The histopathological results (Figure 1D) demonstrated that the liver cells in the NS group were normal in shape and clear in structure, and the lobular cells were arranged radially with the central vein as the center, no inflammatory lesions, and no obvious damage was observed in liver tissue. Compared with the NS group, the hepatic sinuses and central vein of rats were congested in the Arecoline group, the arrangement of hepatocytes was disordered and the boundary was blurred. Nuclear pyknosis, cell gap increased, lymphocyte infiltration, and acidophilic degeneration were also observed in the liver tissue from the Arecoline group. In summary, it can be considered that Arecoline can cause liver damage. 

### 3.2. Metabolomics Study on Arecoline-Induced Hepatotoxicity 

#### 3.2.1. Methodological Investigation 

To ensure the quality, stability, and reliability of metabolomics data, QC samples were used for methodological investigations throughout the entire operation process. Six replicates of the same QC sample solution were prepared, and the RSD values of each ion peak area were calculated. Ions with RSD < 30% accounted for 84.82%, indicating stable and accurate instrument performance. Prepared six QC samples at the same time, and 80.03% of ions with RSD < 30% were obtained, indicating good repeatability of the method. The same QC sample solution was sampled and analyzed at six time points throughout the sampling sequence and calculated the RSD values for each ion. The ions with RSD < 30% accounted for 83.93%, indicating good stability in data acquisition.

#### 3.2.2. Metabolic Profiling Analysis 

**Figure 2:**
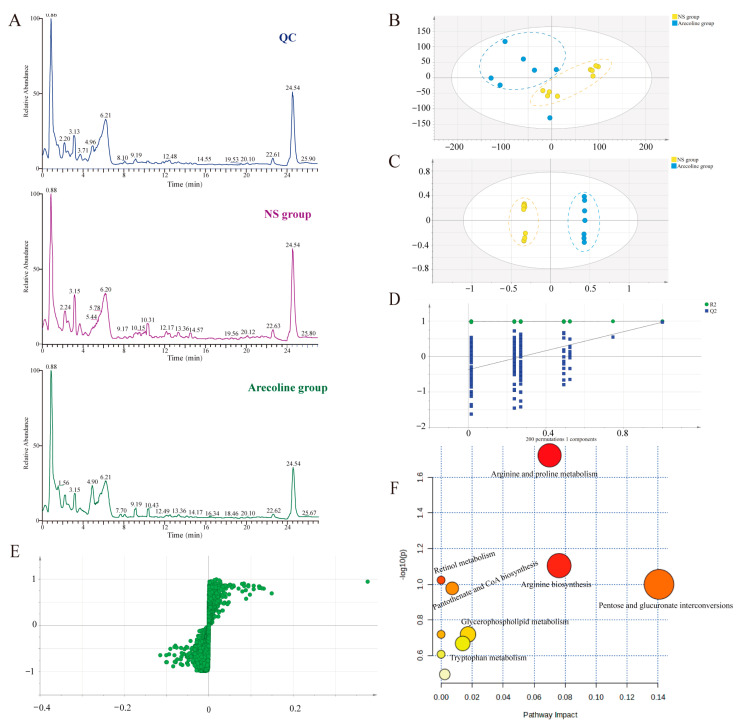
(**A**) TIC chromatogram of serum in the QC sample, NS group, and Arecoline group were obtained based on UPLC-QE-Orbitrap-MS, taking positive mode as an example. (**B**) PCA score plot for NS and Arecoline groups. (**C**) OPLS-DA score plot for NS and Arecoline groups. (**D**) Permutation test of OPLS-DA model. (**E**) S-plots obtained from OPLS-DA model. (**F**) Analysis of metabolic pathways.

Using the UPLC-QE-Orbitrap-MS platform, this research obtained the serum chromatograms of NS, QC, and Arecoline-exposed. The Total Ion Chromatography (TIC) is shown in Figure 2A. In order to explore the effects of Arecoline on the metabolism of rats, principal component analysis (PCA) and orthogonal partial least squares-discriminant analysis (OPLS-DA) were performed on the data. PCA can observe the degree of clustering and dispersion of samples by classifying each group of data. According to PCA (*R*^2^X = 0.77, *Q*^2^ = 0.57) results (Figure 2B), there was a basic separation between the NS group and the Arecoline group, indicating that Arecoline could change the endogenous metabolites of rats. Compared with unsupervised PCA, supervised OPLS-DA can be used to maximize the separation effect and obtain the key variables that lead to differences between groups. As shown in Figure 2C, serum metabolites in the NS group and Arecoline group were completely separated in the OPLS-DA score plot (*R*^2^X = 0.859, *R*^2^Y = 0.988, *Q*^2^ = 0.97), indicating that the endogenous substance in serum changed because of Arecoline-exposed. The existence of overfitting in the OPLS-DA model was verified by two hundred permutation tests. The intercept of the regression line where *Q*^2^ was located on the Y-axis was less than zero, indicating that the model did not have overfitting. This indicated that the model was effective and reliable and could be further used to screen out differential metabolites (Figure 2D). 

#### 3.2.3. Identification of Differential Metabolites 

S-plots were used to determine the differential metabolites after Arecoline administration (Figure 2E) combined with the criteria of VIP ≥ 1 and *p* < 0.05. Identifying possible endogenous metabolites using the mass charge ratio could be completed using the HMDB database. There were 28 differential metabolites between the NS group and the Arecoline group, mainly including D-Lysine, N4-Acetylaminobutanal, L-Arginine, D-Valine, L-Hypoglycin A, Proline betaine, Acetyl-D-carnitine. Table 2 provides pertinent details about the identified differential metabolites in serum.

#### 3.2.4. Metabolic Pathway Analysis 

This research further analyzed the metabolic pathways, which were affected by Arecoline administration using MetaboAnalyst 5.0 online system. The results are shown in Table 3. As shown in Figure 2F, ten metabolic pathways were enriched by the differential metabolites between the NS group and Arecoline group, mainly including Arginine and proline metabolism, Arginine biosynthesis, retinol metabolism, Pentose and glucuronate interconversions, Pantothenate and CoA biosynthesis, biosynthesis of unsaturated fatty acids, glycerophospholipid metabolism, tryptophan metabolism, Aminoacyl-tRNA biosynthesis, and Purine metabolism, among which five metabolic pathways with impact ≥ 0.01 were identified by pathway topological analysis. It can be seen that Arecoline can affect amino acid metabolism, vitamin metabolism, glucose metabolism, and lipid metabolism.

### 3.3. Network Toxicology Study on Arecoline-Induced Hepatotoxicity 

#### 3.3.1. Potential Targets Prediction of Arecoline 

**Figure 3:**
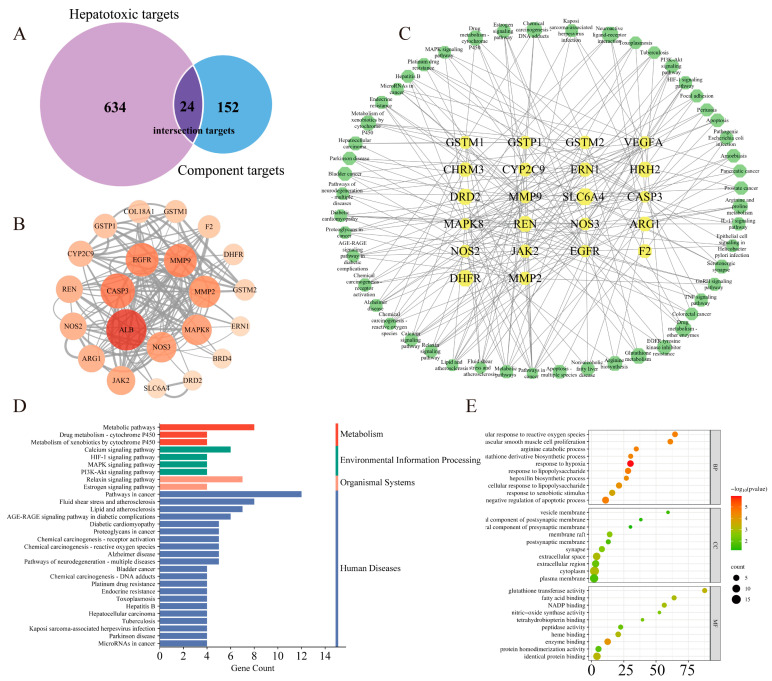
Analysis results of network toxicology. (**A**) Venn diagram of 24 potential common targets were intersected by Arecoline targets and hepatotoxicity targets. (**B**) PPI analysis of common targets. (**C**) “Targets-Pathways” network diagram. (**D**) Enrichment analysis of KEGG pathway. (**E**) GO enrichment analysis.

Based on TCMSP, Herb, Pharm Mapper, and SwissTargetPrediction, 152 targets of Arecoline were filtered out. Meanwhile, a total of 36 hepatotoxicity-related keywords were obtained. Obtained 10,250 “marker/mechanism” genes through searching keywords in the CTD database, and 93,874 “score > 40” genes in the GeneCards database. At the same time, 84 differentially expressed genes were obtained by analyzing the chips (GSE103842, GSE113618, GSE116149, GSE119019, GSE129507, GSE135079) with R language. All the results were integrated and duplicated, and 634 targets of hepatotoxicity were finally obtained. Common targets, intersected by Arecoline targets and hepatotoxicity targets, were regarded as target genes of hepatotoxicity induced by Arecoline. In this study, 24 intersection targets of Arecoline and hepatotoxicity were obtained for further research of toxicity mechanism, and the results are shown in Figure 3A.

#### 3.3.2. Protein–Protein Interaction (PPI) Network Construction 

To search for potential targets in a network further, the interactions of 24 common targets were analyzed using the String database, and the results were visualized using Cytoscape 3.7.2. As shown in Figure 3B, PPI network diagram contains 22 nodes and 144 edges. The value of “Degree” is closely related to the size and color of nodes, and nodes with higher degrees become larger and redder. ALB, CASP3, EGFR, MMP9, MMP2, MAPK8, NOS3, JAK2, REN, NOS2, ARG1, CYP2C9, and GSTP1 are targets for “Degree > 10”.

#### 3.3.3. GO Bioprocess Analysis and KEGG Enrichment Analysis 

DAVID database was used to target enrichment analysis. The KEGG pathway analysis results are shown in Figure 3D, where 51 pathways are enriched, and 30 pathways were selected for visualization based on the *p*-value. GO enrichment analysis results are shown in Figure 3E. A total of 93 biological processes were enriched, mainly “response to hypoxia, “response to lipopolysaccharide”, “negative regulation of apoptotic process”, and “cellular response to reactive oxygen species”. There are 17 cellular components, mainly in “cytoplasm”, “extracellular space”, and “membrane raft”. There are 27 molecular functions, mainly binding reactions, including “enzyme binding”, “glutathione transferase activity”, “identical protein binding”, and “heme binding”.

## 4. Discussion

In this study, on the basis of metabolomics and network toxicology, biomarkers of Arecoline-induced hepatotoxicity in rats were screened, and the potential mechanism was predicted. The specific results are as follows: (1) After continuous intragastric administration of Arecoline, serum biochemical indexes and liver histopathological sections of rats showed a certain degree of liver damage. (2) The results of metabolomics showed that the levels of metabolites such as D-Lysine, N4-Acetylaminobutanal, and L-Arginine changed significantly after administration. (3) The HIF-1 signaling pathway, MAPK signaling pathway, and other related pathways enriched by network toxicology may be the potential pathway for Arecoline-induced hepatotoxicity in rats.

### 4.1. The Manifestation of Arecoline-Induced Hepatotoxicity in Rats 

Arecoline is a pyridine alkaloid in areca nut, which has a wide range of pharmacological activity, such as anti-parasitic, promoting gastrointestinal digestion, analgesic, anti-inflammatory, and anti-allergic. Meanwhile, Arecoline is also a toxic component. There was a study that showed that after a single oral administration of Arecoline in mice, the LD_50_ is 174.7 mg·kg^−1^, and symptoms such as salivation, diarrhea, accelerated breathing, and irritability appear after poisoning [25]. This study suggested that different degrees of toxicity appeared after continuous exposure to Arecoline for 7 days. For example, given the abnormal behavior of the Arecoline group, combined with the “loose stool phenomenon” of the rats, it was preliminarily considered that Arecoline not only had potential hepatotoxicity but also had strong gastrointestinal stimulation and diarrhea, which was consistent with its source (areca nut). Serum biochemical enzyme profile (ALT, AST) also indicated the possible events of hepatotoxicity, and histopathological results clearly revealed that the liver tissue of the Arecoline group showed obvious damage, such as hepatocyte swelling, congestion of hepatic sinuses, lipid degeneration, and inflammatory cell infiltration. The results indicated that Arecoline has a certain degree of toxic effect on the liver, which suggested that the risk of liver damage should be paid attention to when used in large doses.

### 4.2. Network Toxicology Study on Arecoline-Induced Hepatotoxicity 

Based on the results of metabolomics and network toxicology, we found that Arecoline may induce hepatotoxicity by mediating oxidative stress, inflammatory responses, energy and lipid metabolism, and cell apoptosis.

Current research has clearly shown that oxidative stress and inflammatory responses jointly participate in the occurrence and development of hepatotoxic injury [26,27]. Oxidative stress is a state of imbalance between oxidation and antioxidants in vivo. Excessive accumulation of reactive oxygen species (ROS) and reactive nitrogen species (RNS) damages the intracellular antioxidant system and activates inflammatory response. ROS causes inflammatory cell infiltration and the release of inflammatory mediators, resulting in a variety of toxic reactions in tissues and cells [28]. The HIF-1 signaling pathway is one of the most important pathways involved in oxidative stress and mediated inflammatory response. It is mediated and regulated by the hypoxia-inducible factor (HIF) and its regulatory factor von Hippel-Lindau (pVHL) [29]. Previously, research on HIF-1 mainly focused on its active subunit HIF-1α, which is cumulatively expressed under hypoxia conditions and is a key regulator of oxygen content balance in mammals. Research shows that the HIF-1 pathway can regulate ROS by regulating mitochondrial function and changing the degree of electron leakage in the electron transport chain, thus affecting cell survival and proliferation [30,31,32]. In addition, the MAPK signaling pathway enriched by network toxicology is also closely related to liver inflammatory response. The key role of MAPK signaling in drug-induced hepatotoxicity is due to the cascade’s interaction with multiple signaling pathways, as noted in a study [33]. The results of this study suggested that Arecoline can mediate oxidative stress and inflammatory response by regulating JAK, EGFR, MAPK, CASP3, and other key targets and cause hepatotoxicity through HIF-1, MAPK, and other signaling pathways. This was consistent with the conclusion reported in another study that HIF-1, MAPK, and other signaling pathways are the main signaling pathways causing hepatotoxicity [34].

**Figure 4:**
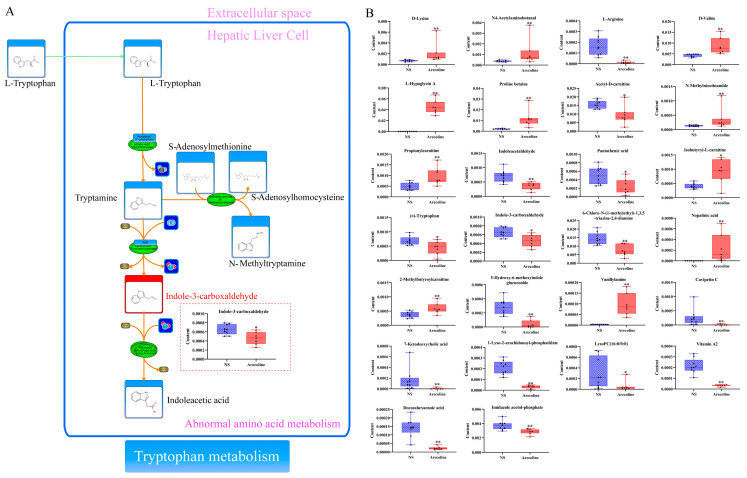
(**A**) Disturbance of tryptophan metabolism. (The red box shows the content of Indole-3-carboxaldehyde in NS group and Arecoline group.) (**B**) Content of differential metabolites, mean ± SEM, * *p* < 0.05, ** *p* < 0.01.

Energy metabolism is one of the most basic characteristics of life, and the body absorbs nutrients from the outside world to maintain life activities through material metabolism. Amino acid metabolism plays an important role in energy metabolism [35,36]. Enzymes that catalyze amino acid transamination, deamination, transmethylation, and decarboxylation are abundant in the liver, which is a key place for the body to metabolize amino acids. Therefore, liver toxicity injury can lead to the abnormal energy metabolism of cells, thus affecting the stability of the amino acid metabolic pathway [37]. Tryptophan is an essential amino acid for the human body, and tryptophan and its metabolites play key roles in a variety of physiological processes. In general, tryptophan has three metabolic pathways, namely the Kynurenine pathway, the 5-hydroxytryptamine pathway, and the indole pathway. Existing studies have confirmed that tryptophan has many biological activities, such as regulating growth, regulating immunity, and anti-oxidative stress [38,39]. In the liver, tryptophan is involved in the synthesis of proteins, and when a toxic injury occurs in the liver, the serum tryptophan level decreases, which in turn promotes the release of pro-inflammatory cytokines and apoptosis, exacerbating hepatic inflammatory response [40]. In this study, (±)-tryptophan, indoleacetaldehyde, indole-3-carboxaldehyde, and 5-hydroxy-6-methoxyindole glucuronide were correlated with tryptophan metabolism, and the contents of these differential metabolites were significantly reduced after the administration of Arecoline (Figure 4). This result indicated that Arecoline might inhibit tryptophan metabolism and lead to a decrease in the content of related metabolites. In addition, Lysine, Valine, Arginine, Glycine, and Ornithine metabolism also play an important role in the body. The metabolomics results in this study showed that the contents of D-Lysine, D-Valine, L-Hypoglycin A, and Nopalinic acid were significantly increased, indicating that the administration of Arecoline promoted the metabolism of these metabolites and increased their contents. In addition, the damage to the mitochondria of hepatocytes can also cause functional abnormalities in some amino acid cleavage enzyme systems, which can hinder amino acid cleavage metabolism [41]. At the same time, the content of other amino acids in serum was also changed, including a significant decrease in the content of L-Arginine, which is involved in processes such as Arginine and proline metabolism, Arginine biosynthesis, and Aminoacyl-tRNA biosynthesis. The decrease in its content indicates that the relevant metabolic pathways are inhibited, which is closely related to energy metabolism, as well as protein biosynthesis and degradation. Carnitine is an important biomarker for determining whether energy metabolism is abnormal. It can provide energy for the body by transporting activated long-chain saturated and unsaturated fatty acids from the cytoplasm to mitochondria, promoting the synthesis of fatty acids β-oxidation. Therefore, the higher the content of carnitine, the faster the oxidation rate of fat and the higher the utilization efficiency of energy. When liver damage occurs, energy metabolism is abnormal, and intracellular mitochondria are destroyed, preventing fatty acid β oxidation, which results in intracellular carnitine accumulation. This is also consistent with the metabolomics results of this study [42]. Serum levels of Propionylcarnitine, Isobutyryl-L-carnitine, and 2-Methylbutyroylcarnitine in the Arecoline group were significantly increased as compared to the NS group. Cytochrome P450 (CYP450) is a type of hemoglobin-coupled monooxygenase that mainly exists in the liver endoplasmic reticulum and plays an important role in the biotransformation of exogenous compounds. Although there are multiple CYP450 subtypes in liver microsomes, only a few enzymes are involved in drug metabolism, one of which is CYP2C9. In the liver, Arecoline interfered with the metabolic process of the CYP450 enzyme system by regulating CYP2C9 and converted cyclophosphamide into its active metabolite acrolein, which further led to the production of reactive oxygen species, caused mitochondrial dysfunction and disrupted the oxidative balance of fatty acids, thus leading to energy metabolism dysfunction and promoting the susceptibility of liver cells to oxidative stress [43]. In addition, retinol metabolism enriched by metabolomics was also associated with the liver. When the liver is injured, retinoic acid could promote activated hepatic stellate cells to enhance the activity of retinol hydrolase in the cells, cause the release of retinol, interfere with lipid metabolism, and affect the homeostasis of energy metabolism [44,45]. In conclusion, the results showed that Arecoline could cause abnormal energy metabolism in hepatocytes and thus lead to hepatotoxicity.

Lipids are important components of cell bilayer membranes, including fatty acyls, glycerol acyls, glycerol phospholipids, and sphingolipids, which have different structures and functions, and lipid homeostasis is crucial for the maintenance of cell morphology, integrity, and function. The key role played by lipids in tissue physiology and cell signaling has been demonstrated in several studies [46], which have shown that abnormal changes in lipid metabolism can lead to cellular dysfunction and necrosis, resulting in cell death. Disorders of lipid metabolism are a key factor contributing to hepatotoxic injury [47,48]. Glycerophospholipid is an amphiphilic molecule that is directly involved in cellular physiological functions and helps transport triacylglycerol. It is one of the most abundant and complex phospholipids in organisms and exhibits significant changes in a variety of diseases. Lysophospholipids are formed by hydrolysis of the fatty acid side chains of glycerophospholipids or sphingolipids, including lysophosphatidylcholine (LysoPC), lysophosphatidylethanolamine (LysoPE), lysophosphatidylinositol (LysoPI), and lysophosphatidic acid (LysoPA). Among them, LysoPC is a highly biologically active molecule involved in inflammatory response and oxidative stress. When liver injury occurs, the expression of cytokine receptors in immune cells, including monocytes, macrophages, and dendritic cells, is inhibited [49]. As the degree of liver injury increases, the content of cytokine decreases, which leads to the decline of liver synthesis, metabolism, and transformation function. The decrease in LysoPC levels may be closely related to the activation of liver inflammatory signaling pathways. The metabolomics results indicated that the serum lipid level of rats changed significantly after Arecoline administration, with the LysoPC (16:0/0:0) and related metabolites such as 7-Ketodeoxycholic acid, Indole-3-carboxaldehyde, 5-Hydroxy-6-methoxyindole glucuronide were significantly down-regulated. The above-mentioned differential compounds are mainly concentrated in glycerophospholipid metabolism, suggesting that Arecoline inhibits glycerophospholipid metabolic processes, thus resulting in a decrease in the content of LysoPC (16:0/0:0) and other metabolites involved in metabolic pathways. In addition, these differential metabolites are also involved in energy metabolism and inflammatory response and are also closely related to liver cell apoptosis [50].

Apoptosis is a spontaneous and orderly death of cells according to their procedures. It has important biological significance and complex molecular biological mechanism and is closely related to the generation of oxygen free radicals, the disturbance of cell energy metabolism, the activation of cytokines, and the expression of Caspase and B lymphocyte tumor-2 family genes [51,52,53,54,55]. Research has shown that the PI3K/AKT signaling pathway is closely related to cell differentiation, proliferation, autophagy, and apoptosis. CASP3 is a member of the Caspase family, which is located downstream of the cascade reaction of Caspas e and is considered to be the only pathway in the apoptosis cascade reaction. Its activation marks the entry of the cell into an irreversible stage of apoptosis [56]. After liver injury, cells may experience severe endoplasmic reticulum stress, leading to excessive activation of Caspase, inhibition of PI3K and Akt acidification, reduction of HIF-1 mTOR expression, disruption of redox homeostasis and energy balance, and promotion of cell apoptosis. In this study, the CASP3 and JAK signaling pathways obtained by network toxicology indicate that Arecoline may induce apoptosis and exhibit hepatotoxicity through them.

### 4.3. Limitations and Prospects of the Study

This study integrated metabolomics and network toxicology to comprehensively analyze the mechanism of Arecoline-induced hepatotoxicity, but further research is still necessary. First, only male rats were used in the toxicological experiments, and the toxicity expression of female rats still needs further verification. Second, the identification of differential metabolites relies entirely on online databases, and the uncertainty of identification may express false positive results, which in turn affects the comprehensiveness of metabolic pathway enrichment. Future targeted metabolomics studies can be further conducted. In the end, the degree of association between enriched pathways and toxicity expression still needs further exploration in vivo and in vitro experiments. Based on the above points, future research will focus on targeted metabolomics and specific mechanism pathways, and further investigate the mechanism of Arecoline-induced hepatotoxicity.

After the Aristolochia acid incident in 2017, the China Association of Chinese Medicine launched the Integrated Evidence Chain (iEC) method for toxicity evaluation. It improves the credibility of the results by integrating multiple research evidence. In this study, Arecoline was the “component evidence” for inducing hepatotoxicity events. At the molecular level, network toxicology and metabolomics, respectively, obtained biomarkers and potential toxic genes of Arecoline-induced liver damage, and explained some toxic mechanisms from genes (upstream) and metabolites (downstream), which are related to “causal evidence”. Pathological sections and serum biochemical indexes detection are objective phenotypic facts and are “result evidence”. In the future, we will obtain more evidence from different levels and provide more research data to elucidate the mechanism of Arecoline’s hepatotoxicity.

## 5. Conclusions

To conclude, this study used metabolomics combined with network toxicology to investigate the mechanism of hepatotoxicity induced by Arecoline. Through metabolomics analysis, the endogenous metabolites in the serum of rats before and after Arecoline administration were analyzed, and differential metabolites such as D-Lysine, N4-Acetylaminobutanal, L-Arginine, D-Valine, and L-Hypoglycin A were obtained. These differential metabolites were mainly involved in lipid metabolism, amino acid metabolism and vitamin metabolism by regulating metabolic pathways such as Arginine and proline metabolism, Pantothenate, and CoA biosynthesis. Based on the network toxicological research strategy of computer simulation analysis, 24 key targets of Arecoline-induced hepatotoxicity were obtained, including ALB, CASP3, EGFR, and MMP9. HIF-1 signaling pathway, MAPK signaling pathway, and PI3K-Akt signaling pathway were obtained through KEGG enrichment. Further investigated and analyzed combining metabolomics and network toxicology results, suggesting that Arecoline may induce hepatotoxicity by mediating oxidative stress, inflammatory responses, energy and lipid metabolism, and cell apoptosis. The results of this study are the data support for the toxicity study of Arecoline and provide a reference for the scientific development and reasonable application of areca nut.

## Figures and Tables

**Table 1:** Dosing regimen of Arecoline hydrobromide.

Grouping	Number	Drug	Dose	Administration Mode	Administration Time
NS	10	0.9% NaCl	8.0 mL/kg/d	p.o., continuous administration	7 Days
Arecoline hydrobromide—low	10	Arecoline hydrobromide	500 mg/kg/d	p.o., continuous administration	7 Days
Arecoline hydrobromide—high	10	Arecoline hydrobromide	1000 mg/kg/d	p.o., continuous administration	7 Days

**Table 2:** Identification results of potential differential metabolites in rat serum.

NO.	RT/min	Metabolites	Obsed *m*/*z*	Calcd *m*/*z*	Error ppm	Formula	HMDB	Content Variance	MS/MS
1	0.705	D-Lysine	147.11280	147.11299	1.29	C_6_H_14_N_2_O_2_	HMDB0003405	↑**	84.08101, 130.08664, 157.62578, 86.99315
2	0.735	N4-Acetylaminobutanal	130.08625	130.08662	2.84	C_6_H_11_NO_2_	HMDB0004226	↑**	84.08099, 130.08662, 56.04992, 72.93741, 100.02428, 112.07578
3	0.819	L-Arginine	175.11895	175.11945	2.86	C_6_H_14_N_4_O_2_	HMDB0000517	↓**	70.06542, 60.05606, 116.07096, 175.11945
4	0.847	D-Valine	118.08626	118.08651	2.12	C_5_H_11_NO_2_	HMDB0250806	↑**	118.08651, 72.08102, 59.07337, 55.05469, 109.05436, 91.05466
5	0.859	L-HypoglycinA	142.08626	142.08662	2.53	C_7_H_11_NO_2_	HMDB0029427	↑**	142.08662, 69.04483, 53.03899, 81.03365, 99.04432
6	0.876	Prolinebetaine	144.10191	144.10190	−0.07	C_7_H_13_NO_2_	HMDB0004827	↑**	60.93843, 59.93072, 98.06031, 99.04432, 116.97234
7	0.932	Acetyl-D-carnitine	204.12303	204.12318	0.73	C_9_H_17_NO_4_	HMDB0240771	↓*	85.02861, 145.04994, 204.12344, 60.08119
8	0.988	N-Methylnicotinamide	137.07094	137.07112	1.31	C_7_H_8_N_2_O	HMDB0003152	↑**	137.07137, 108.04454, 81.07011, 80.04967, 116.96632
9	1.742	Propionylcarnitine	218.13868	218.13911	1.97	C_10_H_19_NO_4_	HMDB0000824	↑**	85.02863, 218.13911, 159.06560, 177.87805
10	1.954	Indoleacetaldehyde	160.07569	160.07610	2.56	C_10_H_9_NO	HMDB0001190	↓**	118.94285, 113.96400, 160.07616, 132.08118
11	2.321	Pantothenicacid	220.11795	220.11812	0.77	C_9_H_17_NO_5_	HMDB0000210	↓*	90.05518, 132.10226, 202.10800, 116.03451, 184.09737, 160.09740
12	2.968	Isobutyryl-L-carnitine	232.15433	232.15439	0.26	C_11_H_21_NO_4_	HMDB0000736	↑*	152.03506, 231.99182, 108.04536, 187.81517, 79.95724, 87.92511
13	3.183	(±)-Tryptophan	227.07910	227.07864	−2.03	C_11_H_12_N_2_O_2_	HMDB0030396	↓*	146.06044, 142.06558, 118.06540, 159.09207, 188.07112, 205.09775
14	3.210	Indole-3-carboxaldehyde	146.06004	146.06047	2.94	C_9_H_7_NO	HMDB0029737	↓*	65.32993, 91.05457, 118.06541, 99.04428, 126.09157, 146.06047
15	3.229	6-Chloro-N-(1-methylethyl)-1,3,5-triazine-2,4-diamine	188.06975	188.07066	4.84	C_6_H_10_ClN_5_	HMDB0033249	↓*	146.06049, 188.07111, 118.06544, 170.06049, 181.06914
16	3.638	Nopalinicacid	263.12376	263.12411	1.33	C_10_H_18_N_2_O_6_	HMDB0029437	↑**	70.06539, 172.07944, 263.12411, 132.10220
17	4.003	2-Methylbutyroylcarnitine	246.16998	246.17030	1.30	C_12_H_23_NO_4_	HMDB0000378	↑**	85.02861, 187.09685, 229.28825, 246.17030
18	4.156	5-Hydroxy-6-methoxyindoleglucuronide	340.10269	340.10281	0.35	C_15_H_17_NO_8_	HMDB0010363	↓**	164.07094, 113.02357, 230.08168, 85.02859, 267.29990, 340.10281
19	5.467	Vanillylamine	154.08626	154.08661	2.27	C_8_H_11_NO_2_	HMDB0012309	↑**	131.97456, 113.96394, 122.06030, 53.03911, 94.06535
20	9.444	CavipetinC	389.26863	389.26941	2.00	C_24_H_36_O_4_	HMDB0030366	↓**	353.24783, 335.23724, 317.22671, 371.25845, 389.26941, 227.14314,
21	9.507	7-Ketodeoxycholicacid	407.27920	407.27884	−0.88	C_24_H_38_O_5_	HMDB0000391	↓**	81.07018, 353.24780, 307.24228, 243.13841
22	13.364	1-Lyso-2-arachidonoyl-phosphatidate	459.25062	459.25089	0.59	C_23_H_39_O_7_P	HMDB0012496	↓**	287.23724, 269.22675, 361.27374, 457.23322, 189.16426, 98.98443
23	14.172	LysoPC(16:0/0:0)	496.33976	496.34085	2.20	C_24_H_50_NO_7_P	HMDB0010382	↓*	184.07381, 104.10723, 86.09664, 313.27386, 60.08119
24	14.692	VitaminA2	285.22129	285.22162	1.16	C_20_H_28_O	HMDB0013117	↓**	241.19538, 267.21115, 129.07010, 131.08566
25	18.113	Docosahexaenoicacid	329.24750	329.24774	0.73	C_22_H_32_O_2_	HMDB0002183	↓**	67.05453, 81.07032, 133.10173, 311.23730, 163.14865, 184.88966
26	24.445	Imidazoleacetol-phosphate	111.01973	111.02024	4.59	C_6_H_9_N_2_O_5_P	HMDB0012236	↓**	88.500332, 97.00977, 100.01137, 115.96432, 133.97482, 126.06840, 138.98064, 111.09196

↑**, significantly increased compared with NS group (*p* < 0.01); ↑*, significantly decreased compared with NS group (*p* < 0.05); ↓**, significantly increased compared with NS group (*p* < 0.01); ↓*, significantly decreased compared with NS group (*p* < 0.05).

**Table 3:** Analysis results of metabolic pathways related to differential metabolites.

NO.	Pathway Name	Metabolite	*p*	−Log(*p*)	Holm p	FDR	Impact
1	Arginine and proline metabolism	L-Arginine; N4-Acetylaminobutanal	0.018907	1.7234	1.0	1.0	0.06998
2	Arginine biosynthesis	L-Arginine	0.07861	1.1045	1.0	1.0	0.07614
3	Retinol metabolism	Vitamin A2	0.094722	1.0235	1.0	1.0	0
4	Pentose and glucuronate interconversions	3-Methoxy-4-hydroxyphenylglycol glucuronide	0.10004	0.99984	1.0	1.0	0.14062
5	Pantothenate and CoA biosynthesis	Pantothenic acid	0.10532	0.97747	1.0	1.0	0.00714
6	Biosynthesis of unsaturated fatty acids	Docosahexaenoic acid	0.19108	0.71879	1.0	1.0	0
7	Glycerophospholipid metabolism	LysoPC (16:0/0:0)	0.19108	0.71879	1.0	1.0	0.01736
8	Tryptophan metabolism	Indole-3-carboxaldehyde	0.21487	0.66783	1.0	1.0	0.0139
9	Aminoacyl-tRNA biosynthesis	L-Arginine	0.24713	0.60708	1.0	1.0	0
10	Purine metabolism	Indole-3-carboxaldehyde	0.32062	0.494	1.0	1.0	0.00234

## Data Availability

Data are contained within the article.
